# Companion Animals as Models for Inhibition of STAT3 and STAT5

**DOI:** 10.3390/cancers11122035

**Published:** 2019-12-17

**Authors:** Matthias Kieslinger, Alexander Swoboda, Nina Kramer, Barbara Pratscher, Birgitt Wolfesberger, Iwan A. Burgener

**Affiliations:** Division of Small Animal Internal Medicine, Department of Companion Animals and Horses, University of Veterinary Medicine Vienna, 1210 Vienna, Austria; Alexander.Swoboda@vetmeduni.ac.at (A.S.); Nina.Kramer@vetmeduni.ac.at (N.K.); Barbara.Pratscher@vetmeduni.ac.at (B.P.); Birgitt.Wolfesberger@vetmeduni.ac.at (B.W.); Iwan.Burgener@vetmeduni.ac.at (I.A.B.)

**Keywords:** cancer models, companion animals, STAT3, STAT5, comparative oncology

## Abstract

The use of transgenic mouse models has revolutionized the study of many human diseases. However, murine models are limited in their representation of spontaneously arising tumors and often lack key clinical signs and pathological changes. Thus, a closer representation of complex human diseases is of high therapeutic relevance. Given the high failure rate of drugs at the clinical trial phase (i.e., around 90%), there is a critical need for additional clinically relevant animal models. Companion animals like cats and dogs display chronic inflammatory or neoplastic diseases that closely resemble the human counterpart. Cat and dog patients can also be treated with clinically approved inhibitors or, if ethics and drug safety studies allow, pilot studies can be conducted using, e.g., inhibitors of the evolutionary conserved JAK-STAT pathway. The incidence by which different types of cancers occur in companion animals as well as mechanisms of disease are unique between humans and companion animals, where one can learn from each other. Taking advantage of this situation, existing inhibitors of known oncogenic STAT3/5 or JAK kinase signaling pathways can be studied in the context of rare human diseases, benefitting both, the development of drugs for human use and their application in veterinary medicine.

## 1. Introduction

Almost half of all households in the United States have at least one companion animal. This means that approximately 77 million dogs and 58 million cats share a common environment with their human owners and are largely exposed to the same health risk factors [1]. In the absence of significant cardiovascular disease, cancer is the number one cause of death for dogs, killing between 40% and 50% of individuals older than 10 years, and between 20% and 25% regardless of age [2,3,4]. Numbers are less detailed for cats, but the overall tumor incidence ranges between 30% and 35% [5]. The prevalence of cancer in companion animals has increased in the last decades, which may be the result of a real increase in cancer incidence, an increase in the population of companion animals at risk or the awareness and willingness of the animal owners to pursue diagnostic and treatment options [6]. While the full spectrum of tumor types seen in humans also occurs in cats and dogs, the rates for individual tumor types are often different. Canine osteosarcoma, soft tissue sarcoma and feline non-Hodgkin’s lymphoma for example are significantly higher than in humans (Table 1), whereas other tumor entities like lung, prostate and colon tumors are rare in companion animals. Cancer in companion animals, particularly in dogs, resembles cancer in humans in many ways, including spontaneous disease occurring without an isogenic background or genetic engineering and shared environmental and societal status with owners. Further similarities in chronology of the disease adapted to lifespan, organization into various well-characterized breeds that show different incidences of tumor types, and shared environmental and societal status with owners makes them attractive objects for comparison.

The mouse has been an extremely useful tool to gain cellular and mechanistic understanding into the development of cancer [17]. The concept of oncogenes and tumor suppressors balancing cellular proliferation of multicellular organisms has been proven in vivo in genetically modified mice [18,19]. The use of mice deleted for a single gene has allowed us to determine the involvement of signaling pathways, genetic regulators like transcription factors, epigenetic factors, etc. in the development and sustained growth of cancer. Overall, experiments in mouse models have been invaluable in understanding the mechanistic basis of cancer biology. However, while offering critical insights into basic concepts, murine models underrepresent the heterogeneity and complex interplay between human immune and cancer cells [20,21].

The Janus kinase (JAK)—signal transducer and activator of transcription (STAT) signaling pathway—provides a fast and efficient way for relaying signals from the extracellular space to the nucleus and modifying gene expression [22]. Main targets of this pathway represent regulators of cell division and apoptosis as broadly discussed in several publications in this special issue [23,24,25]. As these are central aspects in the development of cancer, it is not surprising that members of this pathway are over-activated in many cancers [26,27]. The kinases of this pathway, JAK 1-3 and TYK2 are constantly activated in several different tumor types and are subject to pharmacological inhibition. Recently, also STAT proteins have come into focus of cancer researchers. Particularly STAT3 and STAT5 are activated in over 70% of all human cancer types and constitute a critical node in the signaling networks of tumor cells [28].

This review will highlight, why companion animals and particularly the dog represent an attractive link between murine models, addressing basic mechanistic aspects and human diseases specifically in the context of JAK-STAT signaling.

## 2. Preclinical Models

The vast majority of cancer models currently are represented by mice, and their fundamental importance for preclinical research is clearly established [20,29]. These animals come as various strains that have been inbred over many generations and thereby are genetically highly homogenous [30]. When used for experiments, they are matched for age, sex and size, receive the same sterile diets and are housed under specific pathogen-free (SPF) conditions. All of these factors are controlled for in order to generate standardized conditions that allow us to draw scientifically sound conclusions based on the variation of one single parameter [31]. Furthermore, this reduction of variables and thereby “noise” allows us to reduce the number of animals necessary to reach statistically significant results. While this concept has proven its scientific merits and is logical within itself, it is questionable, whether young, sex-matched and inbred mice on sterile nutrition are a good representation of the typically older, obese and genetically diverse human cancer patients [32].

The controlled environment also affects the outcome of cancer-related experiments. For example, it has become clear recently, that the microbiome influences the response to cancer treatment [33,34]. Accordingly, mice raised under SPF conditions in various research institutions show differences in the composition of their gut microbiome, affecting tumor growth rates [35]. Again, while differences in the gut microbiome are likely to exist in human cancer patients, the single standardized composition under which these experiments are carried out in laboratory mice are very likely no close representation of the much broader spectrum in humans. Additionally, rodents have adopted to most ecosystems metabolically, but in regard to drug metabolism, due to growth-hormone regulated p450 cytochrome components, pharmacodynamic and pharmacokinetic properties, canines are by far superior and therefore used as key models for FDA-drug approved testing [36].

Another important aspect is the genetic background and its modification particularly with respect to the immune system. Mice used for tumor experiments usually are highly inbred, reducing genetic variability [37]. Experiments regarding the consequences of a narrow genetic background have demonstrated potential phenotypic tilting, which may result in unrepresentative biased phenotypes [38]. Furthermore, the buffering of genetic variation, including disease-causing mutations, is impaired under these circumstances [39]. One effort to overcome limitations due to a narrow genetic background is to establish new reference populations derived from the crossing of several different mouse strains, as exemplified by the Collaborative Cross project [40]. By broadening and defining the genetic basis, it offers the perspective of enhancing genetic stability and reproducibility, thereby also representing a new and potentially better resource of murine models for human diseases.

Additionally, and probably of higher practical relevance is the fact that development and proliferation of tumor cells happens in a complex interplay with cells of the immune system [41]. However, cancer-studies in mice are often performed in the absence of a fully functional immune system, using immunocompromised mice as hosts for transplantations of human cell lines, patient-derived xenografts or human tumor-derived immune cell xenografts [42]. The immune system constitutes the major player in the counter-selection against tumor cells, thereby necessitating evasion or adaption strategies on the side of the tumor cells. Since this aspect is missing in such mouse models, the results from experiments thereof likely reflect only partial aspects of tumor biology and challenge their biological relevance.

One way to overcome these problems are humanized mice, which express human instead of murine components of the immune system like major histocompatibility complex, allowing the transplantation of tumor cells in an immunologically at least partially competent environment [43]. These models clearly represent a step forward, however, they are costly, technically complicated and still do not represent all components of a functional and homogenous immune system [44]. Moreover, human patients that have developed cancer are not living under special pathogen-free conditions and are very likely to have chronic viral infections, like the Epstein–Barr virus, cytomegalovirus or herpes simplex, which are present in up to 90% of the total population [45,46,47]. Such chronic infections exert a constant pressure and shape the immune system, which is not the case in mice under SPF conditions. Co-housing of “dirty” outbred immunocompetent mice could be a way for improvement, still mammalian viral species barriers exist, that make companion animals superior in these infectious aspects.

Finally, genetically engineered mice harboring the deletion of a tumor suppressor gene or the ectopic expression of an oncogene or combinations thereof can be used [48]. In this case, the tumors develop in the presence of a competent immune system and problems related to cross-species compatibility do not arise. This setting also enables the introduction of defined mutations that occur in human tumors. However, this comes at the price of costly and time-consuming development, often requiring years of work before availability. Furthermore, whereas the targeted insertion of defined mutations reflecting the human situation is a clear advantage, cancer development is a multi-step process, and the heterogeneity of these further steps is often different between engineered mice and human patients [49,50].

Overall, the mouse has been highly instructive in determining genes involved and their mechanistic contribution to the origin and development of cancer [51]. However, the limitations of this model, in particular the differences in environment and microbiome, life span, tumor etiology and genetic status may be the reason why certain aspects are not reflected closely in this model, resulting in only 11% of oncology drugs that work in mice being approved for human use [52]. Therefore, it is desirable from a translational perspective to add another layer that closer reflects human biology and cancer development.

## 3. Advantages and Disadvantages of Canine Tumor Models

Roughly 4,000,000 dogs and a similar number of cats are diagnosed with cancer each year in the U.S. [53]. Although exact epidemiological data are not available for companion animals, this translates into approximately 5300 cases per 100,000 dogs, which is around ten times higher than in humans with 500 cases per 100,000 persons. This large number of pets provides the opportunity to study spontaneous cancers that are highly similar to those occurring in humans, especially since most pet owners are highly motivated to seek out novel treatments for their companion animals.

There are several advantages of using companion animals as models for human cancer. Among them is the fact that tumors arise spontaneously, just as in humans, and that tumor initiation and progression are influenced by similar factors like age, nutrition, sex, reproductive status and environmental exposure [54]. The risk for developing cancer of the nasal cavity for example is increased up to 60% in animals that are kept by smokers in comparison to pets of non-smoking owners [55,56]. Although there are differences in the diet of humans and companion animals, many components such as meat, vegetables and carbohydrates are derived from the same sources and are consumed non-sterile as opposed to mice under SPF conditions [57]. Furthermore, studies have shown that the contact between owner and pet leads to a large overlap in the microbiome, the importance of which for human tumor development has come into focus recently [58].

Pets, and in particular dogs are large and relatively outbred in comparison to laboratory mice. In fact, the genetic variation across dog breeds or in mixed breeds is similar to the variation in humans on the basis of single nucleotide polymorphisms [59]. In individual pure breeds however, the level of genetic diversity is more restricted [60]. The canine genome has been sequenced with a coverage of 99%, revealing that the approximately 19,000 genes identified in the dog match to homologous or orthologous genes in humans [59]. Actually, for many gene families, particularly for those associated with cancer, the homologies are significantly closer than the relationship between human and mouse [61]. Accordingly, most oncogenes and tumor suppressors that are known from human cancers have been shown to contribute to canine cancers [62].

Dogs and cats of all breeds develop cancer, and the spectrum of cancers seen in companion animals is as diverse as that seen in human patients [63]. The dog is the species in which comparative oncology has shown the most growth, and where it is best characterized [64]. Interestingly, there are breed-specific differences as to the cancer subtypes, reflecting the underlying genetics of the various breeds (Table 2). Mast cell tumors and gliomas for example are over-represented in Boxers, Staffordshire, Weimaraner and Golden Retriever, osteosarcoma in Rottweilers, Greyhounds and Golden Retrievers, bladder cancer in Scottish Terriers, histiocytic sarcomas in Flat-Coated Retrievers and Bernese Mountain Dogs and melanoma and gastric carcinoma in Chow-Chows [65,66,67].

The global expression pattern of canine and human osteosarcoma for example shows a strong similarity, and cluster analysis of orthologous gene signatures does not segregate human and canine tumors [73]. Finally, many chemotherapy protocols used for the treatment of canine cancers have been adopted from human medicine. The same chemotherapeutics used in human lymphoma for example are also active in canine lymphoma (e.g., vincristine, cyclophosphamide, doxorubicin, mitoxantrone, cytarabine and methotrexate), and drugs that are ineffective in human lymphomas are also inactive in canines (i.e., gemcitabine, cisplatin and carboplatin) [5].

One of the biggest advantages of companion animals as models for human cancer is the spontaneous development of tumors in the presence of an intact immune system. Immune cells pose a significant barrier to the development of cancer, and undergo changes themselves, as cancer cells co-opt the immune response [76,77]. As a result, the tumor influences innate as well as adaptive immune cells to become regulatory, rather than tumoricidal [78,79]. This interplay results in the selection of tumor cells that are invisible to anti-tumor T-cell-mediated destruction, and is central for tumor editing and immune evasion. Additionally, similar to humans, dogs with advanced cancer exhibit intrinsic T-cell defects as well as T-cell exhaustion [80]. Immune cell interplay with cancer cells is a JAK-STAT3/5 affair, as detailed in the special issue in several articles [24,25,81].

Like all other model systems, companion animals have strengths as well as weaknesses, both practically and conceptually. The biggest hurdle, when working with cats and dogs in research is the paucity of investigational tools. Many antibodies and recombinant products that are available for humans and mice do not show cross-species reactivity. However, the sequencing of the canine genome and the development of genome editing via the CRISPR/Cas9 technology has relieved many of the restrictions [82]. It is now possible to introduced genetic alterations with high efficiency into any desired locus, facilitating for example the visualization of specific cell types via the expression of marker proteins, or targeted deletion of single genes [83,84]. Conceptually, the lack of standardized housing conditions, making it difficult to control for variables, has been held against non-rodent models. However, as pointed out here, this represents an advantage when it comes to closely mimicking the translational aspects in tumor biology.

**Figure 1:**
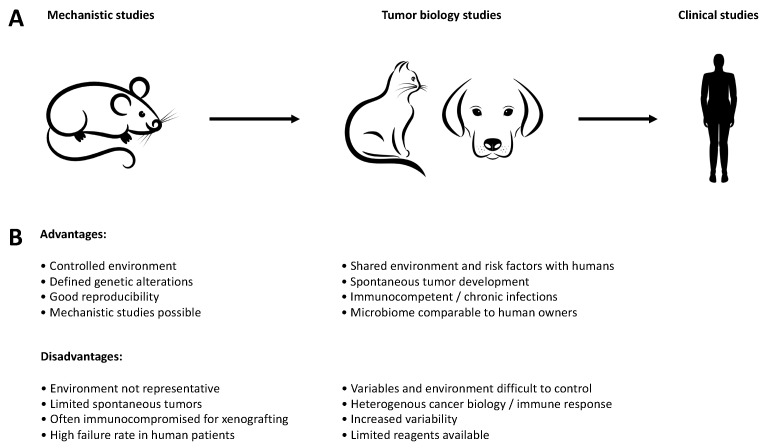
Advantages and disadvantages of different models during drug discovery. (**A**) Companion animals can be used as an intermediate step between the mechanistic work in murine models and clinical studies in humans, particularly with regard to comparative aspects of tumor biology. (**B**) Advantages and disadvantages of the individual models for translation into human clinical studies.

The potential of companion animals as biological models between the basic mechanistic work that is possible in the mouse and translation to humans is demonstrated by 1. the high conservation at the genomic level, 2. the involvement of similar genetic and environmental risk factors, 3. the successful use of canine cancer as biological models for the early development of bone marrow transplantation protocols and 4. canine trials for the development of drug level and exposure durations. This sets an ideal stage to combine new perspectives of targeted therapies and specific molecular inhibitors in the field of comparative oncology for the benefit of human as well as veterinary medicine (Figure 1). Furthermore, this approach is not limited to cancer but applicable to any comparative condition including infectious or inflammatory diseases like inflammatory bowel disease or pre-malignant conditions like adenoma formation or clonal hematopoiesis.

## 4. Relevance and Conservation of the JAK-STAT Signaling Pathway

JAK-STAT proteins constitute an evolutionary conserved signaling pathway [85]. Ligand binding of receptors leads to the activation of JAK kinases and STAT proteins, inducing transcription. The family of JAK kinases consists of four members, JAK1-3 and TYK2, and there are seven highly homologous STAT proteins, STAT1–4, STAT5a and STAT5b and STAT6 [22,86]. For details on the mechanism see also other reviews in this issue [87,88]. As such, this pathway provides a remarkably elegant and straightforward mechanism to transduce signals from receptors to the nucleus.

**Figure 2:**
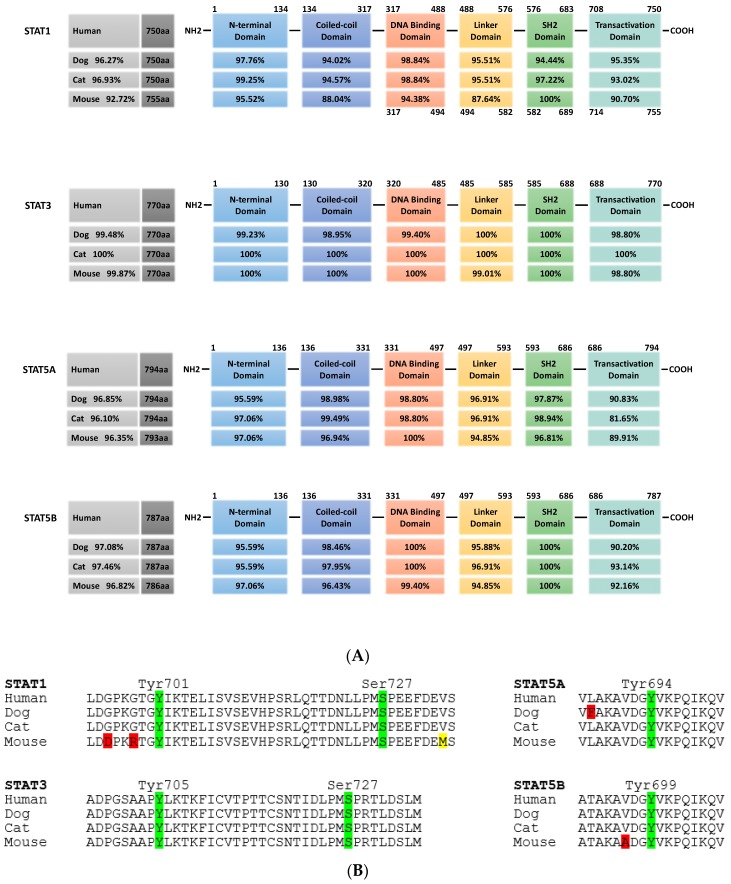
Cross-species conservation of STAT protein domains. (**A**) STAT1, STAT3, STAT5a and STAT5b from dog, cat and mouse are analyzed for their overall homology compared to the respective human protein (grey boxes, left). In the schematic representation of STAT protein domains, the amino acid positions are indicated above. All proteins share the same domain positions, except for murine STAT1, which has a five amino acid insertion in the DNA binding domain (numbers below the scheme indicate the aa position in this case). Percentages in the domain boxes of dog, cat and mouse STAT proteins show the homology of each domain to the human counterpart. Analyses were carried out using ClustalX. (**B**) Comparison of key phosphorylation sites in the transactivation domain of STAT1, STAT3, STAT5a and STAT5b from dog, cat and mouse to the human sequence. Amino acid sequence is shown, with phosphorylation sites in green and position indicated; positive amino acid exchanges (conserving protein function) are indicated in yellow, other exchanges in red. (STAT1: human NP_009330.1, dog XP_848353.1, cat XP_006935505.1, mouse NP_001192242.1; STAT3: human NP_644805.1, dog XP_005624514.1, cat XP_003996930.1, mouse NP_998824.1; STAT5a: human NP_001275647.1, dog XP_548091.2, cat XP_023099834.1, mouse NP_001157534.1; STAT5b: human NP_036580.2, dog XP_548092.1, cat XP_023100377.1, mouse NP_035619.3).

All family members show the same structural organization, i.e., an N-terminal domain required for oligomerization of dimers into tetramers, a coiled-coil domain, a DNA-binding domain, a linker domain, a Src homology 2 (Sh2) domain for dimerization and a C-terminal transactivation domain (Figure 2). Functionally, STAT2, STAT4 and STAT6 regulate specific immune cell responses, whereas STAT1, STAT3 and STAT5 have diverse physiological roles. STAT1 is mostly involved in immunity, host defense against pathogens and cell death, stimulating the transcription of pro-inflammatory and anti-proliferative genes like caspases, *NOS2*, *MDM2*, *CDKN1A* and *CDKN1B*. On the contrary, STAT3 and STAT5 are mostly involved in cell proliferation and prevention of apoptosis, activating the transcription of genes like *CCND1*, *BIRC5*, *c-MYC*, *VEGF*, *MCL1*, *BCL2L1* and *BCL2*. Additionally, STAT3 can also be found in mitochondria, where it supports RAS-dependent malignant transformation via sustained altered glycolytic and oxidative phosphorylation [89,90]. Given their roles in the stimulation of cellular proliferation, the prevention of apoptosis and the stimulation of metabolism, STAT5, and even more so STAT3, are activated in nearly 70% of solid and hematological human tumors [91,92,93].

Silencing or inhibition of STAT3 or STAT5 signaling impairs tumor growth and survival in murine and human studies, while only slightly affecting normal differentiated cells [94,95,96,97]. These findings lead to the concept of STAT3 and STAT5 constituting a “signaling bottleneck” situation for tumor cells, making them attractive targets for inhibition [98]. However, caution has to be exerted with regard to tissue-specificity, as tumor-suppressive functions have been ascribed to STAT3 in neuronal, hepatic and colorectal tumors and to STAT5 in breast cancer [99,100].

Several different ways of inhibiting STAT signaling are possible. Upstream of STAT proteins, JAK kinases are mutated in a broad range of diseases from severe combined immunodeficiency to various forms of cancer, including JAK1 in acute myeloid leukemia, JAK2 in myeloproliferative diseases and JAK3 in different leukemias and lymphomas, and inhibitors against JAK kinases are already approved by the US Food and Drug Administration (FDA) for clinical use [27]. Interestingly, different layers of negative regulators of JAK-STAT signaling are present such as suppressor of cytokine signaling (SOCS), protein inhibitor of activated STAT (PIAS) and protein tyrosine phosphatases, arguing for the necessity of a tightly controlled down-regulation of this signaling pathway [101].

Due to the broad activation, minor side-effects and the overall importance, major efforts by many laboratories and pharmaceutical companies are ongoing to develop inhibitors against STAT3 and STAT5. In both cases, all current inhibitors target one of three STAT motifs: the SH2 domain necessary for the interaction of phosphorylated monomers to form dimers, the N-terminal domain mediating the formation of tetramers from activated STAT dimers and the DNA-binding domain [102]. STAT3 and STAT5 from companion animals show more than 96% homology at the overall protein level to their human counterparts, with a particular high level of conservation of 98% to complete alignment in these three domains (Figure 2). This high level of conservation opens up the possibility to use pet animals as models for diseases in which the JAK-STAT signaling pathway is over-activated.

A good example for such a successful application is already established. Cytokine dysregulation has been implicated in allergic skin disease, particularly in atopic dermatitis in humans. T-helper cells type 2 (Th2) produce increased levels of IL4, IL5, IL10, IL13 and IL31, in addition to elevated production of IFNγ by T-helper cells type 1 (Th1), signals that all converge on the JAK-STAT signaling pathway [27,103,104,105]. Dermatological problems are the second most common reason for dogs to present to veterinary practices, frequently including allergic skin diseases like atopic dermatitis [106,107]. In the skin of atopic dogs, a cytokine profile can be found that resembles the human condition, and in an experimental model of canine allergic dermatitis, elevated transcripts of IL6, IL13 and IL18 and IFNγ were detected, supporting the idea that cytokine dysregulation plays a role in allergic skin disease [108,109,110]. The novel JAK inhibitor oclacitinib is most potent against JAK1, but also affects JAK2 and JAK3 at reduced efficiency, inhibiting the function of JAK1-dependent inflammatory cytokines [111]. Treatment of dogs suffering from atopic dermatitis results in a reduction of associated skin lesions and oclacitinib recently has been approved in the US and Europe for the treatment of allergic/atopic dermatitis [112,113,114]. Overall, inhibition of JAK1-dependent cytokines is an effective and novel way to treat canine allergic skin disease, proving the high similarity and cross-species conservation of JAK-STAT signaling.

## 5. Inhibition of STAT3 and STAT5 in Companion Animals: Current Status/Future Perspectives

Currently, STAT3 and STAT5 are studied in several cancer types of companion animals. Canine mammary cancer cells and diffuse large B cell lymphoma as well as feline oral squamous cell carcinoma and mammary tumors show activation of STAT3 or STAT5. First results indicate reduced proliferation and increased apoptosis upon inhibition of JAK1/2 or STAT3 respectively [115,116,117,118,119,120,121]. Two areas exemplify the potential of research and development concerning STAT proteins and human—canine comparative oncology best [122,123]. Osteosarcoma is the most frequent form of malignant bone disease in dogs, however, it is relatively rare in humans. The estimated incidence rate is at least 13.9/100,000 in canines and 1.02/100,000 in humans, affecting primarily children [124,125]. Such a high incidence rate provides a good opportunity to study a rare human disease using dogs as a preclinical model. Moreover, canine and human osteosarcoma share many key features like tumor location, early metastasis, development of chemotherapy-resistant metastases and altered expression or activation of several proteins [126].

Consistent activation of STAT3 occurs in a large subset of human and canine osteosarcoma and osteosarcoma cell lines, but not in normal osteoblasts. Down-regulation of STAT3 expression or activity reduces proliferation and induces apoptosis in human and canine cell lines [127,128,129,130]. Additionally, human and canine osteosarcoma possess overlapping transcriptional profiles, further supporting the concept that these diseases are similar at the molecular level [131]. Spontaneous canine osteosarcoma has been used for the development of novel therapeutics such as muramyl tripeptide, IGF-1R inhibitors and rapamycin [132]. However, despite aggressive treatment, 30–40% of children and >90% of dogs are still dying from disease, demonstrating the need for new therapeutic options. The similarities between human and canine osteosarcoma, together with the dependency on JAK-STAT signaling, particularly STAT3, make osteosarcoma one of the prime areas for comparative oncology studies, especially since a large number of STAT3 inhibitors are currently being developed.

Mast cell tumors arise from the uncontrolled proliferation of transformed mast cells mostly in skin, spreading primarily to spleen, liver and bone marrow [133]. While mast cell tumors are relatively rare in humans, they are frequent in dogs, accounting for 7–21% of cutaneous tumors [134,135]. Metastasized tumors in humans as well as in canines have a poor prognosis and short survival times [133,134,136]. Stem cell factor and its receptor c-KIT are essential for mast cell survival and inhibition of apoptosis, and gain-of-function mutations are present in human and canine mast cell tumors [137,138,139,140,141,142]. Accordingly, two inhibitors of c-KIT, masitinib and toceranib have been approved for use in c-KIT driven mast cell tumors in dogs. Both drugs are able to suppress tumor growth temporarily, however, relapses are high, indicating the need for the identification of further targets and therapeutic options [143,144].

STAT1, STAT3 and STAT5 are activated down-stream of mutant c-KIT, but only STAT5 is transcriptionally active in neoplastic mast cells [145,146]. Inhibition of JAK2 and STAT5 was recently discovered to inhibit proliferation and survival of canine mastocytoma cell lines, identifying JAK2/STAT5 signaling as a new potential target in mast cell tumors [147]. Therefore, an attractive option is to use the frequent occurrence of canine mast cell tumors to determine if inhibitors of STAT5 can be used alone or in combination with inhibitors of c-kit or other kinases as a new therapeutic option. Indeed, the potential of STAT5 inhibitors to overcome resistance to a multi-kinase inhibitor in neoplastic mast cells has been demonstrated [148]. Consequently, the next step is to determine the potential of new STAT5 inhibitors in vivo [149]. Both of these examples, osteosarcoma as well as mast cell tumors offer thereby the possibility of using new pharmaceutical in companion animals as a closer biological mimic of the human situation.

## 6. Conclusions

Taken together, cancer in dogs resembles cancer in humans in many ways, like its latency, clinical manifestation and metastatic behavior, its pathobiological characteristics like tumor heterogeneity, its genomic instability and pharmacogenomic signatures including chemoresistance and last but not least its multifactorial nature, including genetic and environmental risk factors [150]. The inability of murine cancer models to recapitulate certain aspects of human tumors is increasingly recognized and illuminates the huge potential of spontaneous canine and, to a lesser extent, feline cancer [151,152,153]. The JAK-STAT pathway is activated in the vast majority of solid and hematological tumors, and is necessary for tumor growth and prevention of apoptosis. The major efforts that are ongoing to develop inhibitors specifically for STAT3 and STAT5 can be extended to studies in companion animals, an option that is particularly attractive for rare human diseases occurring more frequently in dogs and/or cats.

## Figures and Tables

**Table 1:** Incidence rates of various tumor types from human, dog and cat.

Tissue	Human	Dog	Cat
Mammary	127.5 [7]	250 [8]	13–25 [9]
Melanoma	22.2 [7]	19.8 [10]	ND
Testes	5.9 [7]	16.7 [11]	ND
Connective Tissue	3.5 [7]	40.1 [10]	17.0 [1]
Skin	98.85 [12]	103.3 [10]	34.7 [1]
Oral	11.3 [7]	20.4 [1]	11.6 [1]
NHL/Leukemia	33.7 [7]	76.3 [13]	41 [14]
Bone	1.0 [7]	27.2 [15]	3.1–4.9 [16]

Numbers represent cases per 100,000. ND = not determined.

**Table 2:** Oncological disposition of various dog breeds.

Breeds	Most Frequent Tumor Types
Bernese Mountain Dog	Histiocytic sarcoma [68], Lymphoma [68,69], Osteosarcoma [68]
Boxer	Glioma [67,69], Mast cell tumor [10,67]
Flat-Coated Retriever	Soft tissue sarcoma, Histiocytic sarcoma, Hemangiosarcoma [70]
Golden Retriever	Mast cell tumor, Lymphoma, Oral Melanoma, Fibrosarcoma [67]
Magyar Viszla	Mast cell tumor, Hemangiosarcoma, Lymphoma [10,71]
Giant Schnauzer	Epidermal tumor, Hair follicle tumor, Melanocytic tumor [10]
Airedale Terrier	Melanoma [72], Lymphoma [67], Prostatic carcinoma [67]
Bullmastiff	Mast cell tumor, Lymphoma [67]
St. Bernard	Lymphoma [67], Osteosarcoma [73]
Irish Wolfhound	Osteosarcoma [67,74], Lymphoma [75]

The most frequent tumor types of dog breeds with high tumor incidence.
